# Case report: A study on the *de novo KMT2D* variant of Kabuki syndrome with Goodpasture’s syndrome by whole exome sequencing

**DOI:** 10.3389/fped.2022.933693

**Published:** 2022-08-26

**Authors:** Shuolin Li, Jing Liu, Yuan Yuan, Aizhen Lu, Fang Liu, Li Sun, Quanli Shen, Libo Wang

**Affiliations:** ^1^Department of Respiration, Children’s Hospital of Fudan University, Shanghai, China; ^2^Department of Cardiovascular, Children’s Hospital of Fudan University, Shanghai, China; ^3^Department of Rheumatology, Children’s Hospital of Fudan University, Shanghai, China; ^4^Department of Radiology, Children’s Hospital of Fudan University, Shanghai, China

**Keywords:** Kabuki syndrome, *KMT2D* gene, whole exome sequencing, Goodpasture’s syndrome, missense variant

## Abstract

Kabuki syndrome (KS) is a rare genetic disorder characterized by dysmorphic facial features, skeletal abnormalities, and intellectual disability. *KMT2D* and *KDM6A* were identified as the main causative genes. To our knowledge, there exist no cases of KS, which were reported with pneumorrhagia. In this study, a 10-month-old male was diagnosed to have KS with typical facial features, skeletal anomalies, and serious postnatal growth retardation. Whole exome sequencing of the trio family revealed the presence of a *de novo KMT2D* missense variant (c.15143G > A, p. R5048H). The child was presented to the pediatric emergency department several times because of cough, hypoxemia, and anemia. After performing chest CT and fiberoptic bronchoscopy, we found that the child had a pulmonary hemorrhage. During research on the cause of pulmonary hemorrhage, the patient’s anti-GBM antibodies gradually became positive, and the urine microalbumin level was elevated at the age of 12-month-old. After glucocorticoids and immunosuppressant therapy, the patient became much better. But he had recurrent pulmonary hemorrhage at the age of 16 months. Therefore, the patient underwent digital subtraction angiography (DSA). However, the DSA showed three abnormal bronchial arteries. This single case expands the phenotypes of patients with KS and Goodpasture’s syndrome, which were found to have a *de novo KMT2D* missense variant.

## Introduction

Kabuki syndrome (KS; OMIM #147920) is a rare, multiple malformation syndrome characterized by distinctive facial features combined with skeletal abnormalities, immunological defects, and intellectual disability (1, 2, 3). The prevalence of KS is around 1 in 32,000 live births (1).

The major causes of KS are variants in lysine methyltransferase 2D (*KMT2D*) and lysine demethylase 6A (*KDM6A*) (4, 5). *KMT2D* (also called *MLL2*, *ALR*, or *MLL4*) encodes a histone methyltransferase protein that contains 5,537 amino acids in length and methylates the LYS-4 position of histone H3 (6). Studies using the Xenopus model system found that loss-of-function of *Kmt2d* impedes neural crest development (7). *KDM6A* (formerly known as *UTX*) is a well-known epigenomic regulator that interacts with KMT2D and encodes a protein that eliminates the trimethylation of histone 3 lysine 27. Pathogenic *KDM6A* variants can cause KS (8).

Kabuki syndrome has been associated with several genetic variants. In this case report, we described a single case with clinical characteristics compatible with KS. We used a whole exome sequencing to find a *de novo* missense variant in *KMT2D* that explained the phenotype.

Goodpasture’s syndrome, which is often used synonymously to refer to anti-glomerular basement membrane (GBM) disease, is a severe and extremely rare autoimmune disorder characterized by the presence of circulating autoantibodies directed against the non-collagenous domain of the α3 chain of type IV collagen (9, 10). The majority of patients were present with features of rapidly progressive glomerulonephritis and 40% to 60% have concurrent alveolar hemorrhage because of antibodies deposited in both glomerular and alveolar basement membranes (10). Very few studies of childhood Goodpasture’s syndrome are available. We reported a 10-month-old male baby with KS and pulmonary hemorrhage. Whole exome sequencing (WES) of his trio family revealed a *KMT2D* missense variant (NM_003482: exon 48: c.15143G > A, p. R5048H). During the search for the cause of pulmonary hemorrhage, we found that pulmonary hemorrhage in the patient was caused by Goodpasture’s syndrome, which is rare in a patient with KS.

## Case presentation

This study was approved by the Ethics Board of the Children’s Hospital of Fudan University. Written informed consent was obtained from the patient’s parent before submission. The CARE guidelines were followed in the reporting of our case.

A 10-month-old boy was delivered at 35 and 6/7 weeks *via* normal spontaneous vaginal delivery. His mother and father aged 33 and 31 years, respectively, and were non-consanguineous. The boy’s birth weight was 3,145 g, and his height was 49 cm. The child was admitted to the NICU ward as he was suffering from aspiration pneumonia and neonatal necrotizing enterocolitis. At the time of birth, no facial or phenotypic abnormalities were described. Anal stenosis deformity was found during NICU hospitalization, and atrial septal defect (ASD) and ventricular septal defect (VSD) were found using echocardiography. At 21 days of age, he underwent an anoplasty and perineal fistula repair because of congenital anal stenosis and rectal perineal fistula. Afterward, he had VSD repair, patent ductus arteriosus amputation, and patent foramen ovale repair under cardiopulmonary bypass at the age of 4-month-old. The patient has feeding difficulties after birth, easy choking, and spitting up, hence, supplementary food was added at 7 months old. The child lagged in growth and development could not turn over until 10 months. His family history was unremarkable.

**FIGURE 1:**
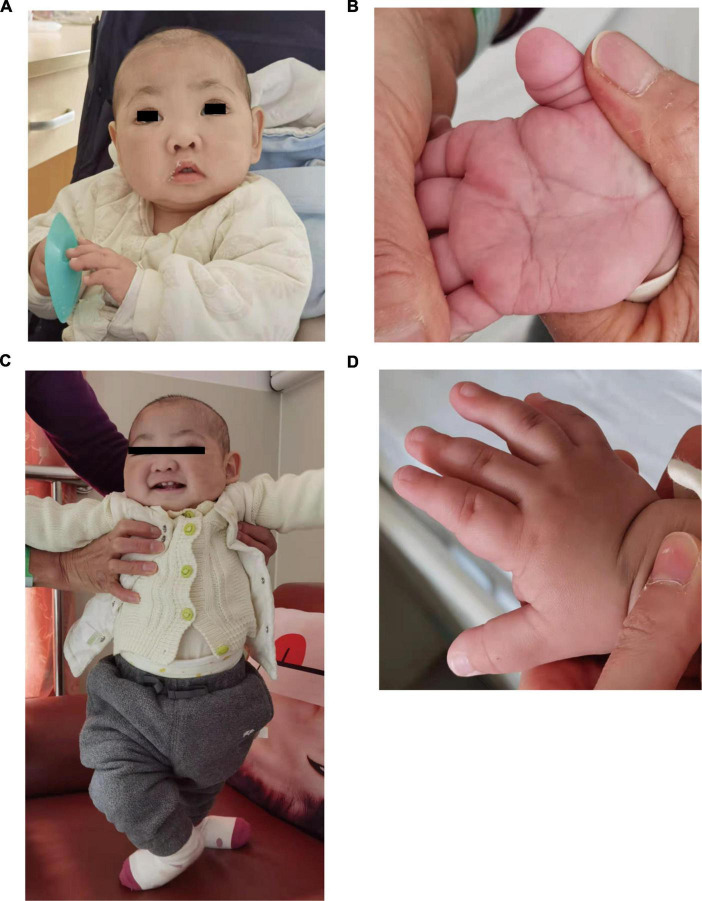
Photographs of the patients in this study. **(A,B)** Showed makeup appearance. **(C,D)** Showed short fingers.

At 10 months of age, he visited our hospital due to pneumonia with anemia for the first time. His chest X-ray showed exudation of both lungs with interstitial changes, and the hemoglobin decreased from 11.0 to 6.4 g/dl. After antibiotic treatment, transfusion of red blood cells, and intravenous immunoglobulin support, his chest x-ray became better and hemoglobin rose to 11.0 g/dl, therefore, he was discharged home. His facial examination showed arched eyebrows, long palpebral fissures with lateral eversion of the lower eyelids, inverse epicanthus, wide nasal bridge, lower lip concave, and large ears (Figures 1A,C). Other characteristics, including brachydactyly and prominent fetal finger pads, are shown in Figures 1B,D. In addition to these facial phenotypes, the patient also had post-natal growth deficiency, motor delay, joint hypermobility, hypotonia, and feeding problem. He had abnormal dentitions and an abnormal genitourinary system due to which he underwent surgery. A head computed tomography (CT) showed that part of the extracerebral space was widened, bilateral ventricles were full, and there were multiple low-density shadows on the skull. Abdominal ultrasonography showed no abnormalities. Abdominal CT suggested the possibility of stones in the right kidney.

**FIGURE 2:**
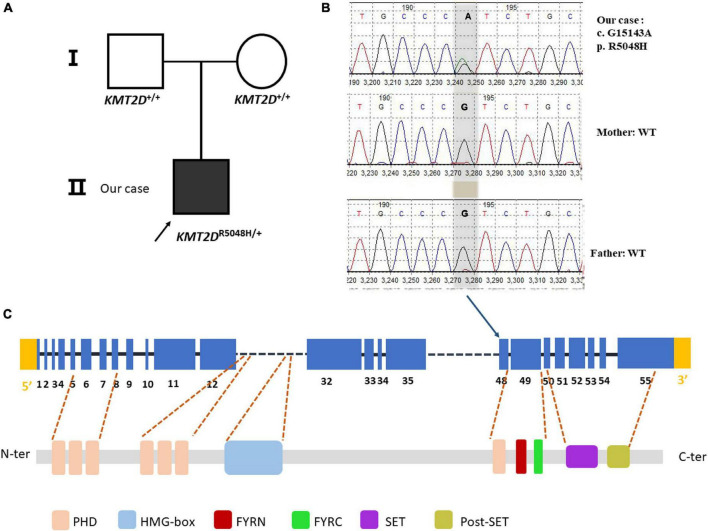
Sanger sequencing of the case family. **(A)** Showed pedigree of the family. **(B)** A heterozygous G > A substitution was confirmed by sanger sequencing at position c.15143, changing an arginine codon into a histidine codon at amino acid position 5,048 (p.R5048H). **(C)** The variant is at the exon 48 of the *KMT2D* gene (NM_003482.3) but not at any domain of the KMT2D protein. PHD, plant homeotic domain; HMG-box, high mobility group-box; FYRN, FY- rich N-terminal domain; FYRC, FY-rich C- terminal domain; SET, Su (var) 3-9, enhancer-of-zeste and Trithorax domain.

Our patient showed typical characteristics of KS as mentioned. We performed WES on the trio family. The patient showed a missense variant of c.15143G > A (p. R5048H) in exon 48 in the *KMT2D* gene. The variant was compared with dbSNP,^1^ 1000 Genomes,^2^ as well as Exome aggregation consortium (ExAc) database.^3^ The variant was not reported as polymorphism and was neither found in ExAC nor 1000G. It is a known disease mutation in Human Gene Mutation Database (HGMD CM 138442) which has been reported in Makrythanasis et al’s (11) study. Sanger sequencing confirmed that the child’s parents did not carry the variant, so we determined it as a *de novo* heterozygous variant (Figure 2). Meanwhile, the variant c. 15143G > A was predicted to be probably a disease caused by Polyphen-2^4^ and MutationTaster.^5^ According to the ACMG guideline (12), the pathogenesis of the variant c.15143G > A (p. Arg5048His) should be pathogenic strong (PS1).

After a short time, at 12-month-old, the patient was admitted to our pediatric pneumology department after recurring fever (37.9–39.4°C), cough accompanied by tachypneic, tachycardia, severe dyspnea, blood from the nasogastric tube, and anemia (hemoglobin level, 7.6 g/dl). At the time of hospital admission, the physical findings were reported as follows: body weight: 7.1 kg (<3rd percentile); body temperature: 37.9°C; blood pressure: 86/58 mmHg; heart rate: 136 beats/min; respiratory rate: 38 breaths/min; oxygen saturation at room air: 76%. He was dyspneic and withdraw blood from the nasogastric tube. Laboratory tests revealed anemia, a mean corpuscular volume of 80.3 fl, a reticulocyte count of 3.9%, and serum iron around 5.4 μg/dl. Ferritin (113.8 μg/L) and total iron binding capacity (49.2 μmol/L) were normal. The C-reactive protein was found to be increased (26.92 mg/L; normal value, <8 mg/L). Liver and kidney function indexes were within the normal range. Serologic tests for autoimmune diseases were negative on 9 August 2021. After several times screen, the anti-glomerular basement membrane antibody (anti-GBM) was shown to be high (71.1 RU/ml; normal value, <20 RU/ml) while urine microalbumin was elevated (NAG/CR 12.59, normal range, 0.3–1.2; IGGU/CR 40.7, normal range, <14; A1MU/CR 615.3, normal range, <14; ALBU/CR 152.9, normal range < 26.5). Oxygen with high-flow nasal cannulae was administered and a prompt blood transfusion was performed. Intravenous infusion of cefotaxime (100 mg/kg/day, 3 times daily for 8 days) was also performed.

**FIGURE 3:**
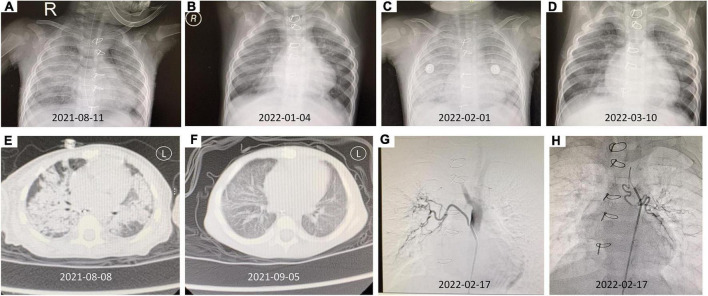
The imaging findings of this patient. **(A)** Chest X-ray (2021-08-11) showed a massive, diffuse bilateral large pulmonary consolidation. **(B)** The chest X-ray after therapy (2022-01-04). **(C)** The chest X-ray (2022-02-01) suggested extensive exudate. **(D)** After abnormal bronchial artery was embolized, the follow-up chest X-ray (2022-03-10) was significantly better than before. **(E)** Chest CT (2021-08-08) revealed increased patchy and flaky shadows were evident in both lungs, and a local thickening of bilateral pleura was seen. **(F)** The chest CT after therapy showed better than Figure 3E (2021-09-05). **(G)** The DSA showed right bronchial artery originated from the thoracic aorta. **(H)** The DSA showed left bronchial artery originating from the aortic arch.

Chest X-ray showed a massive, diffuse bilateral large pulmonary consolidation (Figure 3A). Chest CT showed increased patchy and flaky shadows, which were evident in both the lungs; with this, local thickening of bilateral pleura was seen (Figure 3E).

To find the reason why this patient has anemia and pulmonary hemorrhage, fiberoptic bronchoscopy was performed early after admission showing that active diffuse bleeding was objectified and there existed no foreign body in the bronchus and tumor. A bronchoalveolar lavage (BAL) revealed increasingly hemorrhagic returns and abundant hemosiderin-laden macrophages. The etiologic assessment was conducted, which was not contributive, except revealing the presence of *Hemophilus influenzae* in BAL culture and identification of bocavirus from the virus test. Isotope lung inhalation test and gastroscopy that had been performed showed no apparent abnormality. Cardiac ultrasound indicated no regurgitation after ventricular defect repair, bicuspid aortic valve, and pulmonary hypertension (36 mmHg).

After multidisciplinary consultation and discussion with the pediatric rheumatology, cardiology, respiratory, and imaging departments, the diagnosis of Goodpasture’s syndrome was performed. Immunosuppressive therapy was initiated with high-dose methylprednisolone (30 mg/kg per day for 3 days) and intravenous immunoglobin supporting therapy for 3 days. During the hospital admission, his respiratory symptoms have been improved, and his hemoglobin raised to 10.7 g/dl. A review of autoimmune antibodies showed that anti-GBM antibodies were negative, and urinary microproteins were significantly reduced. Hence, plasmapheresis has not been indicated. The patient was discharged in good general conditions with the following drugs: Prednisone 2 mg/kg/day, 1 time a day; Mycophenolate mofetil dispersible tablets 30 mg/kg/day (0.125 g), two times a day. The patient was in good general condition at the follow-up examination. The chest X-ray and chest CT have shown better than before (Figures 3B,F). At the moment, the patient is in good condition, being treated with oral corticosteroids.

During the outpatient follow-up, the child again developed anemia and dyspnea at the age of 16-month-old. The chest X-ray suggested extensive exudate (Figure 3C) with a drop in hemoglobin (7.3 g/dl). At the same time, the anti-GBM antibody showed a value of 24.4 RU/ml. Considering the possibility of repeated pulmonary hemorrhage, combined with the fact that the child is with KS, and has a history of VSD and ASD, digital subtraction angiography (DSA) is recommended to check whether there is abnormal pulmonary circulation. After undergoing DSA, it was observed that the left bronchial artery originates from the aortic arch, the right bronchial artery originates from the thoracic aorta, and the left and right trunk inferior phrenic arteries originate from the right renal artery (Figures 3G,H). Abnormal bronchial artery embolization was done successfully.

After discharge, the patient (weight 8.5 kg) was given oral methylprednisolone 16 mg, 1 time a day, and mycophenolate mofetil dispersible tablets 0.25 g, 1 time every 12 h, and iron, calcium supplementation, and omeprazole to prevent corticosteroids side effects. The follow-up chest X-ray examination was significantly better than before (Figure 3D), the hemoglobin was elevated (10.2 g/dl), and there were no symptoms of dyspnea as found recently.

## Discussion

A *de novo* missense variant in the *KMT2D* gene was described in a boy with distinctive facial features and pulmonary hemorrhage diagnosed with Goodpasture’s syndrome. The patient showed classical clinical features of KS, including arched eyebrows, long palpebral fissures with lateral eversion of the lower eyelids, depressed nasal tip, large ears, lower lip concave, ASD, VSD, postnatal growth retardation, hypotonia, intellectual disability, poor feeding and nutrition, congenital anal stenosis, and rectal perineal fistula.

To date, KS is caused due to pathogenic variants in *KMT2D* or *KDM6A* genes (13, 14). Variants in the *KMT2D* gene have been most frequently identified and are present in 55–80% of KS (15). Different *de novo KMT2D* variants have been reported in sporadic patients with KS (16). In familiar cases, an autosomal dominant inheritance has been observed. We identified a missense *de novo* variant in exon 48 of *KMT2D* (c.15143G > A, p. R5048H). This variant was not reported from the 1000 Genomes Database and the Exome Variant Server, suggesting that it is not a common variant. This variant c.15143G > A was also found in Makrythanasis et al’s (11) study in *KMT2D* (also known as *MLL2*) variant detection done on 86 patients with KS (17). Consistent with our study, Banka et al. (18) have also shown that the pathogenic missense variants were commonly located in exon 48. Although this variant does not belong to any domain of the KMT2D protein, it was predicted to be probably a disease caused by Polyphen-2 and MutationTaster. According to the ACMG guideline (12), the pathogenesis of the variant c.15143G > A (p. Arg5048His) should be pathogenic strong (PS1).

In the laters years of childhood, patients with KS develop an immune dysfunction, including susceptibility to infection and autoimmune disorders (19). Studies about immune deficiency in KS demonstrated that low immunoglobulin levels were a more common manifestation (20, 21) so many cases are susceptible to infections, including recurrent otitis and pneumonia (22). In addition, the most common autoimmune disease were hematological disorders, such as autoimmune hemolytic anemia, idiopathic thrombocytopenic purpura, and autoimmune hepatitis (19, 22, 23). Matsushima et al. (24) have reported the first patient with KS and pernicious anemia. However, our understanding of the mechanisms underlying autoimmune disorders related to KS is limited. In this study, we review the immunological phenotypes of KS as shown in Table 1. So far, no patient with KS has been reported with Goodpasture’s syndrome.

**TABLE 1:** Immunological phenotypes of KS in pediatric patients in literature.

References	Infection susceptibility	Hypogamma-globulinemia	↓ IgA	↓ IgG	↓ IgM	Autoimmune disease	ITP	AIHA	AITD	VT	AIN	Others
Di Candia et al. (21)	2/5	2/5	5/5	3/5	2/5	–	–	–	2/5	0/5	–	–
So et al. (29)	3/21	0/21	0/21	–	–	–	2/21	1/21	1/21	–	–	–
Margot et al. (19)	57/134	31/58	–	–	–	13/134	6/134	4/134	0/134	6/134	–	–
Lindsley et al. (22)	9/13	3/13	9/13	5/13	4/13	–	–	–	–	1/13	1/13	AIH 1/13
Stagi et al. (23)	51/59	–	36/54	21/51	–	–	17/59	7/59	3/59	4/59	2/59	–
Armstrong et al. (30)	–	2/48	–	–	–	–	1/48	–	–	–	–	–
Hoffman et al. (20)	–	–	15/19	8/19	2/19	3/19	–	–	–	–	–	–
White et al. (31)	14/27	–	–	–	–	–	–	–	–	–	–	–
matsumoto et al. (32)	73/116	1/116	–	–	–	–	1/116	1/116	–	–	–	–
Wessels et al. (33)	114/24	–	–	–	–	–	–	–	–	–	–	–

“–” Not reported;↓ decreased.

AIHA, autoimmune hemolytic anemia; AITD, autoimmune thyroid disease; AIN, autoimmune neutropenia; AIH autoimmune hepatitis; ITP, Immune thrombocytopenic purpura; VT, vitiligo.

Goodpasture’s syndrome is a severe and extremely rare autoimmune disorder characterized by the presence of circulating autoantibodies directed against the non-collagenous domain of the α3 chain of type IV collagen (9, 10). Antibodies found in both glomerular and alveolar basement membranes resulting in glomerulonephritis (GN) and acute pulmonary hemorrhage are usually from the IgG class, with IgG1 and IgG3 subclasses predominating, while rare cases of IgA- and IgG4-mediated disease have been reported (10, 25, 26). Unlike reports in adults, very few cases of childhood Goodpasture’s syndrome are available. In Menzi’s retrospective analysis, pulmonary fundings are not present commonly before puberty and the minimum age at diagnosis is 2 years old (27). However, the age of symptom onset in our case report was 12-month-old, which may be related to KS.

Abnormal persistence of germinal centers, along with a defect in class switch recombination and reduced antibody production was seen in *Kmt2d* knockdown mice (28). Missense variants in the terminal region of the *KMT2D* gene may increase the risk for autoimmune disorders (22). Laboratory tests showed that patients with *KMT2D* variants displayed defective B cell differentiation (22, 24). A defective B cell differentiation may lead to humoral immune deficiency and autoimmune disorders. Although we cannot determine whether Goodpasture’s syndrome is part of the KS phenotypic spectrum or if it occurs coincidentally, this case report does expand on the phenotype of KS and the possible associated autoimmune disorders. Current treatment protocol by the Hammersmith group remains at the cutting edge of therapy. Patients with Goodpasture’s syndrome should be started on prednisolone (1mg/kg tapered over 6–9 months) and cyclophosphamide for 2–3 months in combination with daily plasmapheresis for 14 days or until the anti-GBM antibody is no longer detectable (10). These drugs decrease the immune system’s production of antibodies. Although after immunosuppressive treatment, the patient’s antibody declined rapidly, anti-GBM antibody levels, pulmonary hemorrhage condition, and renal function must be monitored at regular intervals.

## Data availability statement

The datasets for this article are not publicly available due to concerns regarding participant/patient anonymity. Requests to access the datasets should be directed to the corresponding author.

## Ethics statement

The studies involving human participants were reviewed and approved by the Ethics Committee of Children’s Hospital of Fudan University. Written informed consent to participate in this study was provided by the participants’ legal guardian/next of kin. Written informed consent was obtained from the minor(s)’ legal guardian/next of kin for the publication of any potentially identifiable images or data included in this article.

## Author contributions

SL and JL collected the data and wrote the manuscript. LW revised the manuscript. YY collected the patient information. AL, FL, LS, and QS diagnosed the disorder, performed the experiments, and emended the manuscript. All authors read and approved the final version of this manuscript.

## Footnotes

1
http://www.ncbi.nlm.nih.gov/projects/SNP/

2
http://www.1000genomes.org

3
http://exac.broadinstitute.org/

4
http://genetics.bwh.harvard.edu/pph2/

5
http://www.mutationtaster.org/
